# Antibody Aggregation: Insights from Sequence and Structure

**DOI:** 10.3390/antib5030019

**Published:** 2016-09-05

**Authors:** Wei Li, Ponraj Prabakaran, Weizao Chen, Zhongyu Zhu, Yang Feng, Dimiter S. Dimitrov

**Affiliations:** 1 Protein Interactions Section, Cancer and Inflammation Program, Center for Cancer Research, National Cancer Institute, National Institutes of Health, Frederick, MD 21702, USA; chenw3@mail.nih.gov (W.C.); zhuzhongyu@mail.nih.gov (Z.Z.); fengya@mail.nih.gov (Y.F.); dimitrdi@mail.nih.gov (D.S.D.); 2 Intrexon Corporation, Germantown, MD 20876, USA; praba.ponraj@gmail.com

**Keywords:** monoclonal antibodies, antibody aggregation, protein unfolding, antibody domains, antibody drug conjugates

## Abstract

Monoclonal antibodies (mAbs) are the fastest-growing biological therapeutics with important applications ranging from cancers, autoimmunity diseases and metabolic disorders to emerging infectious diseases. Aggregation of mAbs continues to be a major problem in their developability. Antibody aggregation could be triggered by partial unfolding of its domains, leading to monomer-monomer association followed by nucleation and growth. Although the aggregation propensities of antibodies and antibody-based proteins can be affected by the external experimental conditions, they are strongly dependent on the intrinsic antibody properties as determined by their sequences and structures. In this review, we describe how the unfolding and aggregation susceptibilities of IgG could be related to their cognate sequences and structures. The impact of antibody domain structures on thermostability and aggregation propensities, and effective strategies to reduce aggregation are discussed. Finally, the aggregation of antibody-drug conjugates (ADCs) as related to their sequence/structure, linker payload, conjugation chemistry and drug-antibody ratio (DAR) is reviewed.

## 1. Introduction

The tertiary structure of a protein with a given amino acid sequence is defined by competing molecular-scale interactions, which balance the contributions of fold-favoring interactions, such as electrostatic attraction, hydrophobic interaction and hydrogen bonding, to those of unfolding-favoring interactions, like the geometric constraints of chemical bonds, the avoidance of steric clash and electrostatic repulsion [1]. Under native conditions, proteins fold quickly (in a time frame of μs to ms [2]) from the unfolded state (U) to the folded structure (F) in a cooperative manner with several short-lived, meta-stable intermediates (molten globule states) in the down-hill free energy landscape [3]. Proteins experience constant sampling between the folded and partially-folded structures [4]. Under stress wherein either the folded state (F) destabilizes (G_F_ increasing and ΔG_U-F_ decreasing) or the (partially) unfolded structure becomes stabilized (G_U_ decreasing) [5], proteins could populate partially unfolded conformations and result in aggregation. Protein aggregation is a process in which protein molecules self-associate with each other. Non-native aggregation forms via the strong non-covalent contacting of protein molecules and is thermodynamically stable and irreversible [6]. Aggregation requires proteins to experience unfolding or partial unfolding to present key stretches of residues (so-called “hot spots”) to achieve strong interactions between monomers, which often constitutes a rate limiting step before nucleation growth for aggregation [7]. In many cases, protein aggregates use the energetically-favorable β sheets as the building modules [8]. A common example for this kind of aggregation is amyloidosis, in which the polypeptides build fibril aggregates by stacking against β sheets along the fibril axis [9].

mAbs-based pharmaceuticals have enjoyed increasing success in therapeutic markets [10] and typically target high impact areas, such as cancers, infectious diseases, auto-immune diseases and metabolic disorders [11]. mAbs bear many therapeutic metrics, such as high binding affinity and specificity, long circulation half-life in blood stream, non-toxic nature and easy manufacturing. Although highly desired, one bottleneck limiting mAbs therapeutics’ development is aggregation [12,13]. mAbs with 12 sub-domains, large hydrodynamic radii and surface areas, non-symmetrical hydrophobicity and charge distributions are prone to aggregation [14,15]. The immunoglobulin Greek-key β sandwich folding of mAbs is susceptible to edge-edge association [16]. Besides, complementarity determining regions (CDRs) of mAb responsible for antigen binding can also contribute to aggregation due to the frequent occurrences of hydrophobic and electrostatic residues [17,18]. Furthermore, the extensive hydrophobic patches on the surfaces of mAbs, especially on Fc could mediate aggregation [19,20]. These aggregation propensities are amplified by the natural bivalency of mAb. Importantly, the aggregation of mAb could be increased when administered by subcutaneous (SC) delivery in a high mAb concentration of >100 mg/mL [21]. At such high concentrations, mAbs are more susceptible to aggregation [22]. Furthermore, antibody aggregation is highly undesirable, because it could compromise biological functions [12], induce immune responses by breaking B-cell tolerance [23,24] and evoke antibody clearance machinery in vivo [25]. These disadvantages make the control of antibody aggregation imperative in the route to developing successful therapeutics.

Although the mechanism underlying antibody aggregation is generally not fully understood, the aggregation propensity for a given mAb is a function of solution conditions, such as temperature, pressure, pH, ionic strength and excipients (osmolytes, surfactants). Formulation optimization is commonly used to reduce aggregation [26,27]. On the other hand, the susceptibility to aggregation is pre-defined by the intrinsic properties of the antibody, including primary sequence and tertiary structure. Protein aggregation needs some degree of conformational distortion or partial unfolding of the native monomer to expose the aggregation-prone residues to form strong inter-molecular interactions [28]. Antibodies usually contain more than one aggregation-prone region (APR) [29]. Therefore, by contrast to some simple proteins, in which aggregation occurs in a high cooperativity manner [30], antibody aggregation usually occurs through several intermediate states [31], indicating that multi-domain antibody unfolding and aggregation could be understood by analyzing the aggregation of individual domains. The studies of aggregation mechanisms and resistant strategies for antibody domains have inspired the related research of full-length antibodies, although the results obtained from the antibody domains could not always be transferred to the full-sized antibodies. In this paper, we review the impact of sequences and structures on the aggregation of both full length antibodies and antibody domains while discussing aggregation resistance strategies through rational designs.

Antibody-drug conjugates (ADCs) are an important class of therapeutics in oncology. Two ADCs have been recently approved by the U.S. Food and Drug Administration (FDA), including Kadcyla (ado-trastuzumab emtansine) for the treatment of breast cancer and ADCETRIS (brentuximab vedotin) for combating relapsed Hodgkin’s lymphoma. ADC is produced by conjugating a toxic reagent to mAb by chemical reactions, which often destabilizes the conformations of mAbs due to the decreased interchain disulfide bonds and the exposed hydrophobic patches. In addition, the hydrophobicity of the linker payloads could expand the APRs, facilitating the aggregation of ADCs. Thus, ADCs are believed to be more prone to aggregation than the parent mAbs. The elucidation of the aggregation behaviors and mechanisms of ADC could help to conquer the aggregation hurdles in ADC development. In this review, we also discuss the aggregation of ADC by focusing on the impact of the drug-to-antibody ratio (DAR) on aggregation.

## 2. Protein Aggregation

### 2.1. Why Does a Protein Aggregate?

**Figure 1:**
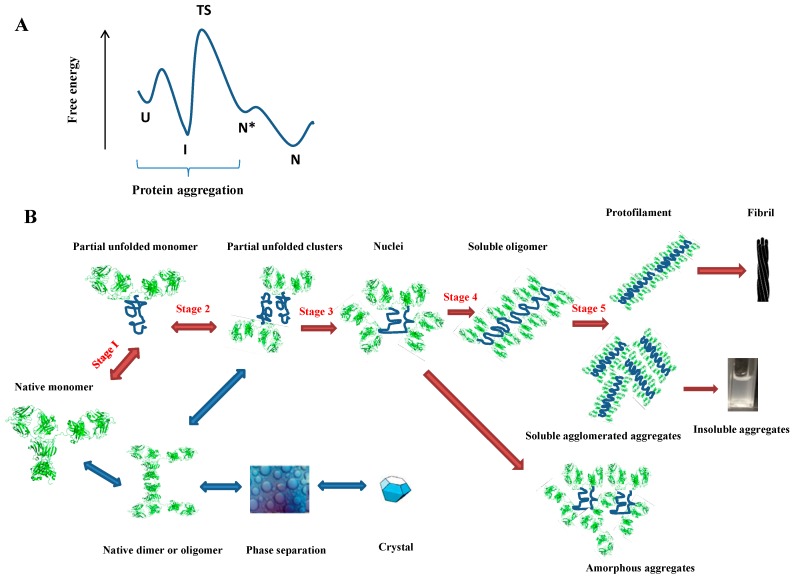
The process of protein folding and aggregation. (**A**) Folding is presented based on the classical thermodynamic and kinetic principles. U, I, N*, N, TS refer to the unfolded state, partially-folded state, locally unfolded state, folded state and transient state, respectively. The conformational ensembles of U, I, N* are prone to aggregation; (**B**) Schematic representation of the protein aggregation process and the possible involved intermediates. This figure uses a monoclonal antibody as an example, but the general behaviors and principles are also applied to other proteins. The red arrows represent the non-native aggregation, while the dark blue arrows denote the native aggregation. The bidirectional arrows show the reversible steps, and the mono-directional arrows account for the irreversible process.

The native protein has the lowest free energy and the most stable conformation [3] (Figure 1). This is maintained by a spectrum of fine interactions: hydrogen bonds of the main chains and side chains; van der Waals and hydrophobic interactions; the constraints of energetically-unfavorable bond torsions and steric clashes; maximizing chain entropy; electrostatic repulsion and attraction; and interactions between amino acids and the solvent [32]. These interactions collectively balance the protein conformations [33]. Under conditions in which the folded states overwhelm the unfolded states, protein monomers populate the native conformations. In such a case, the nature of the energy barriers for the transient state (TS) prevents protein ensembles from populating aggregation-prone states, and thus, the protein remains soluble without a risk of misfolding and aggregation [34]. However, these energy barriers are compromised under stressing conditions, wherein the decreased G_TS_ combines with the increased G_F_ kinetically and thermodynamically shifts the “on-way folding” pathway to the “off-way aggregation” pathway, allowing proteins to experience an irreversible aggregation route [5].

### 2.2. How Does a Protein Aggregate?

In general, the aggregation process mediated by folding intermediates could be divided into the following five stages (Figure 1B): (I) partial unfolding of the native monomer; (II) reversible self-association of the partially-unfolded or folded protein; (III) net irreversible aggregation nuclei formation; (IV) further aggregation growth by monomer chain addition; (V) aggregation association to form the high molecular weight soluble aggregates or insoluble precipitation [35].

Protein aggregation mediated by folding intermediates is triggered by the unfolding of native monomer. The unfolding energy barrier (ΔG_U-F_) that allows the samplings of partially-disordered conformations needs to be overcome in Stage I of aggregation [28]. ΔG_U-F_ not only intrinsically correlates the protein associated G_U_ and G_F_, but also depends on temperature (T), pressure (*p*) and the conditions of the solution [36]. Stage II involves the association of unfolded monomers, which is governed by the protein colloidal interactions implicated by the hydrophobicity and charge distributions of proteins [37,38]. The key stage for protein aggregation lies in Stage III, the nucleation step, in which the unfolded protein experiences the structural re-arrangement, such as the alteration of surface charge distributions, the exposure of the aggregation-prone regions (mainly referring to the hydrophobic patches to the solvent) and changes of the topologies for the β sheet regions or even re-orientation of the α-helix into the β-strand [5]. This step usually constitutes the rate-limiting step in the protein aggregation, after which the subsequent growth stages are much faster [7]. The existence of this nucleation stage is rationalized by the experimentally-observed “lag time” during protein aggregation, which could be shortened by “seeding” the preexisting aggregates [39]. The overall protein aggregation occurs kinetically rather than dynamically and, therefore, is pathway dependent [7]. Thus, it is often experimentally observed that proteins bear good thermostability (low G_F_), but indeed aggregates readily due to kinetically-favorable conditions [40].

### 2.3. How to Mitigate Protein Aggregation?

Although protein aggregation is a priori unpredictable kinetically, the thermodynamic aggregation potentials could be inferred from the intrinsic characteristics of protein sequences and structural features [7,18]. Therefore, aggregation could be well controlled by the rational design of protein sequences and structures. Protein unfolding and the nucleation are usually the key steps for protein aggregation, and the subsequent monomer addition and aggregate association occur much faster. Thus, strategies ameliorating protein aggregation often target the aggregation Stages I, II and III. The corresponding methods for mitigating aggregation thus are: (1) stabilizing the native monomer (decreasing G_F_) or destabilizing the partially-unfolded monomer (increasing G_U_) to reduce the potential of protein unfolding at Stage I; (2) altering the protein surface charge distributions to increase the electronic repulsion between the unfolded monomers at Stage II; and (3) disturbing the structural re-arrangements of unfolded monomers in Stage III to disfavor hydrophobic contacts and the packing of β strands. These strategies could be learned from the nature of protein structures and mechanisms. For example, many proteins involved in hereditary forms of protein deposition diseases bear mutations decreasing the conformational stability of the folded monomer and promote aggregation in vitro [38,41,42]. In addition, the native proteins disfavor the sequences of alternating polar and non-polar residue for β strand assembly [43], as well as clusters of many consecutive hydrophobic residues to decease the tendency of aggregation before folding [44]. Antibodies either inwardly point a charged residue in the middle of the β strand to disfavor the hydrophobic associations or they locate a proline to introduce a bulge dissecting the strand to avoid edge-edge association [16].

### 2.4. Computational Methods for Studying or Predicting Protein Aggregation

Protein aggregation involves various aggregate intermediates and pathways. Many computational models have emerged to dissect the aggregation mechanism and to evaluate how the external factors, pH, ionic strength, etc., influence the aggregate intermediates. In this regard, many groups exploited the coarse-grained (CG) lattice models to study the aggregation mechanism, in which protein molecules are treated as on-lattice single chains, and residues represented are as beads [45,46]. Their interactions are calculated in specifically pre-defined force fields, and the molecular association equilibrium is simulated using conformation searching algorithms, such as Monte Carlo (MC). To obtain more kinetical details, many groups have performed the more accurate atomistic simulations by simplifying proteins into the peptides bearing high aggregation propensities. These molecular simulations provide insights into exploring the oligomeric conformations that can seed the aggregation [47] and figuring out how peptide side chains kinetically and dynamically affect the amyloid aggregation [48].

On the other hand, many computational algorithms have been developed to predict APRs, which usually have unique sequences regarding charge, aromaticity, hydrophobicity and secondary structural propensity [49,50]. These calculation tools could be classified into two types. One is the statistical algorithms to rank the propensity of aggregation for stretches of amino acids by comparing them to the existing polypeptide databases composed of amyloidogenic peptides, which includes Aggrescan (Aggregation Scan), PAGE (Prediction of Aggregation), TANGO (a statistical mechanics algorithm), Zyggregator, Amylpred (Amyloid Prediction), etc. [18,51,52]. Most of these tools only use the protein sequence as input to find out the short APRs with 5–9 residues prone to forming amyloid-like fibrils. Although these methods have achieved some success, one should be cautious about the APRs identified by these methods, since these APRs are just necessary, but not sufficient for forming aggregates. To facilitate aggregation, APRs need to be exposed to contacting the neighboring molecules. Therefore, APRs prediction should be verified by the experimental results when the APRs do not exist in the solvent exposed loops or on the surface of folded proteins. The other method for identifying APRs is the molecular simulation (MD), which ranks protein conformations according to their aggregation propensity. Cecchini et al. have used MD to predict APRs in human amyloid β-peptide, amylin and the prion protein PrP Ure2p_1-94_ [52]. Recently, Chennamsetty et al. have developed a full atomistic MD simulation method (spatial aggregation propensity (SAP)) to identify aggregation-prone motifs with surface exposed hydrophobic residues on full IgG1 [53]. By using SAP, they have successfully achieved mitigating the aggregation of IgG1 by mutating those APRs [54]. Collectively, these calculation methods could provide convenient methods for ranking protein candidates in the early development process, which could guide the design of aggregation-resistant proteins.

## 3. Antibody Aggregation

### 3.1. Sequences and Structures of mAbs

**Figure 2:**
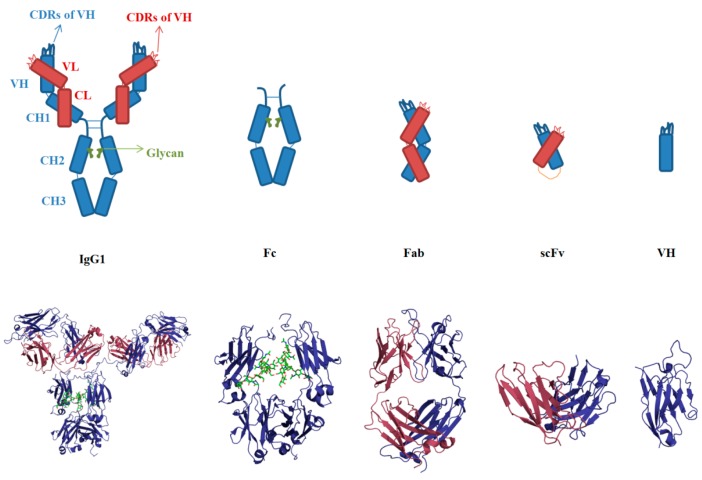
Molecular architecture of an immunoglobulin G1 (IgG1) antibody and its fragments. An IgG consists of two heavy chains (blue) and two light chains (red). The glycan is presented by the green color. Fc (the crystallizable fragment) is a dimer of C_H_2, C_H_3 and glycans. Fab (antigen-binding fragment) is composed of variable heavy (V_H_) and light (V_L_) domains, as well as two constant domains (C_H_1 and C_L_). ScFv is the artificial format containing V_H_ and V_L_ connected by a flexible linker (yellow). V_H_ (or V_L_) is the minimal unit for antigen binding mediated by complementarity determining regions (CDRs). The CDR loops in the V_H_ domain are denoted as H1, H2 and H3 (blue); the CDRs in the V_L_ domain are named as L1, L2 and L3 (red). Below are the 3D structures of an HIV neutralizing antibody b12 with intact IgG1, Fc, Fab, scFv and V_H_ formats.

The overall architecture of a typical IgG consists of two identical light chains and heavy chains. Each light chain folds into two domains, V_L_ (variable light) and C_L_ (constant light), while each heavy chain contains four domains of V_H_ (variable heavy), C_H_1, C_H_2 and C_H_3 (Figure 2) [55]. The whole IgG forms three structural units of equivalent size, two Fabs and a Fc dimer. Each Fab is composed of V_L_, C_L_, V_H_ and C_H_1, and Fc is a homodimer of C_H_2 and C_H_3. Fab and Fc are loosely connected by the hinge region and are not considered to interact with each other. These multi-domains enable the IgG to allocate its functions into different domains. Both V_H_ and V_L_ in Fab collectively mediate the antigen binding via CDR loops. Fc is involved in effector functions and pharmacokinetics by interacting with receptors.

**Figure 3:**
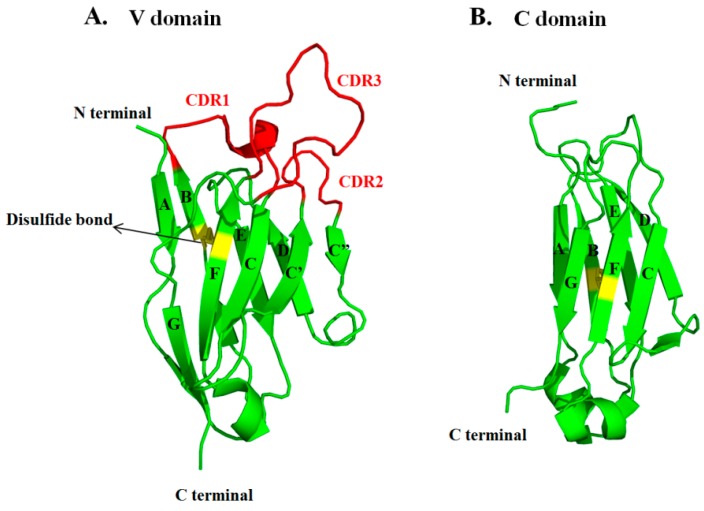
The structural comparisons of the V domain and the C domain. (**A**) and the C domain (**B**). The β sheets are presented by the green cartoon models with the CDRs denoted in red and the disulfide bond in yellow. The anti-parallel β sheets are numbered as A, B, C, C’, C’’, D, E, F. The C domain does not contain the C’ and C’’ strands.

The V domains of IgG are the smallest entities for antigen binding. The V domain consists of two layers of β sheets connected by a disulfide bond and by a cross-over connection through the CDR1 loops (Figure 3) [56]. The C domain lacks C’ and C’’ strands, which would otherwise correspond to CDR2 in the V domain. The CDR3 loops in V_H_ and V_L_ are established by the V-D-J and V-J rearrangement of the antibody gene respectively and somatic mutations that contribute to high diversities for targeting various antigens. CDRs are the core part for antigen binding, which contain high frequencies of aromatic and hydrophobic residues, such Tyr, Phe, Leu and Ile, as well as residues functioning as hydrogen bonding donors, such as Ser, Thr, Asn and Gln [18]. V_H_ and V_L_ associate with each other via hydrophobic interactions involving residues Val37, Leu45 and Trp47 (according to Kabat numbering). C_H_1 and C_L_ hold together by strong hydrophobic packing between residues Val190, Phe174 and Leu143 in C_H_1 and Leu135, Phe116, Phe118 and Val133 in C_L_, which combined the V_H_/V_L_ association to constitute the stable Fab [31]. Fab connects to Fc via the flexible hinge region, where the upper region contains several disulfide bonds for establishing inter-chain ligation, and the lower region is usually hydrophobic and participates in the Fc receptor binding. The flexibility of the hinge ensures the domain movement and the orientation of Fab and Fc, which could modulate the antigen binding and effector function. C_H_2 is believed to be the least stable domain in IgG due to the lack of direct interactions between the C_H_2 dimer, except the weak side chain interactions of glycans [57]. In contrast, the C_H_3C_H_3 dimer comes into tight contact with each other by the hydrophobic interactions involving residues Tyr438, Phe436, Leu391 and Leu372 and electrostatic interactions, such as salt bridge of Glu357-Lys370 and Asp399-Lys409 [58]. C_H_2 associates with C_H_3 via the salt bridges of Lys248-Glu380 and Lys338-Glu430. Collectively, IgG forms a well-folded globular structure via extensive intra-domain and inter-domain interactions.

### 3.2. Aggregation of Full-Length IgG

IgG contains extensive intra-domain and inter-domain hydrophobic interactions. When subjected to structural fluctuations, those hydrophobic interactions are readily exposed to constitute aggregation nuclei [59]. The subdomains of IgG belong to the immunoglobulin superfamily (IgSF) with β-strand sandwich folding, which is intrinsically prone to amyloid aggregation by edge-edge association [18]. For example, trastuzumab (Herceptin) contains two closing β-strands in the Fab, wherein one strand (SVFIFP) at the edge of the four-stranded β-sheet of C_L_ is packed against the four-stranded β-sheet of the C_H_1 domain, thus mediating aggregation [18]. The intermolecular beta sheet associations were frequently found in the IgG1 aggregate induced by various stress conditions, such as heating and stirring [60]. Besides those non-covalent associations, the free sulfhydryl group derived from the impaired disulfide bonds of IgG promotes aggregation through intramolecular scrambling and/or intermolecular crosslinking [61]. In this regard, particular attention needs to be paid to the unpaired non-canonical Cys for antagonizing aggregation. Buchanan et al. have achieved decreasing the aggregation propensity of Ang2 mAb by mutating a free Cys residue in the proximity of light chain CDR2 (LCDR2) (Cys49) into Thr or Asn [62]. On the other hand, the aggregation susceptibility of IgG is deeply modulated by the features of CDRs. Given that CDRs are also responsible for antigen binding, it is challenging to engineer the CDRs to resist aggregation without compromising antigen binding. To reconcile the antigen binding, aggregation-resistant engineering usually does not directly target CDR bearing the APRs, but rather the edge residues flanking CDRs or the surrounding regions. Wu et al. have improved the solubility of an anti-IL-13 monoclonal antibody CNTO607 by mutating a set of hydrophobic residues (Phe-His-Trp) in heavy chain CDR3 (HCDR3) to Ala. However, the binding affinity of the mutated mAb was decreased significantly (>1000-fold) [17]. Alternatively, instead of engineering HCDR3, the authors have resorted to introducing a hydrophilic glycan into the neighboring HCDR2, which could shield the hydrophobic triad in HCDR3, but meanwhile did not intervene in the antigen binding [17]. Another elegant study came from Dudgeon et al. showing that the incorporation of negatively-charged residues, such as Asp and Glu, into the HCDR1 and LCDR2 did not impact the antigen binding and function of full-length trastuzumab against HER2, probably because it is HCDR3 and LCDR3, rather than HCDR1 and LCDR2, that contribute to HER2 binding [63].

Another factor complicating IgG aggregation is the glycosylation. Usually, the glycans attached at N297 are believed to benefit the aggregation resistance for IgG by shielding hydrophobic residues from being exposing to the solvent. Trout et al. have used SAP calculations to demonstrate that glycan attached at IgG1 Asn297 shields the couples of hydrophobic residues, such as F241 and F243, from exposure to the solvent. Consequently, the aglycosylated mAbs are less stable and therefore aggregate more easily than the glycosylated mAbs [64]. In addition, it is believed that glycoforms could adjust the conformation of Fc in either “open” or “closed” states and, thus, modulate the colloidal interactions between IgG [65]. Schaefer et al. have reported that IgG bearing high mannose derived from yeast is more resistant to aggregation than the counterpart expressed by mammalian cells with complex type glycans [66]. Hence, one can change aggregation liability by altering the glycoform of IgG or introducing additional glycans on IgG [67]. By contrast, the hydrophobic patches in Fc, which constitute the docking sites for other molecules to implement important biological functions, are “hot spots” for aggregation [68]. Trout et al. have exploited SAP to identify the exposed hydrophobic residues spreading across the whole sequences of Fc, such as Leu309 in C_H_2 and Ile253 in the C_H_2–C_H_3 junction [53]. Those hydrophobic patches have been reversed by introducing the mutations of L234K, L235K, I253K and L309K to decrease IgG–IgG self-association and aggregation [54]. Although the different IgG isotypes bear relatively conserved constant fragment and relatively invariant hydrophobic patches, they have intrinsically distinct thermostabilities and colloidal stabilities due to the minor differences of the Fc sequences, the length of the hinge linker, the number of disulfide bonds in hinge regions and the pattern of glycosylation [69,70]. In many cases, it is believed that IgG1 has the highest stability compared to IgG2, IgG3 and IgG4. IgG2 has two more cysteines in the hinge compared to IgG1 and is prone to the presentation of free sulfhydryl radicals for exacerbating aggregation. IgG3 has a relatively long hinge region, which is susceptible to protease cleavage and renders IgG3 prone to chemical degradations. IgG4 is prone to forming into the bi-specific dimer by domain swapping [71]. Thus, most therapeutic mAbs have adopted the IgG1 forms. However, in special cases, the aggregation propensity of IgG1 could be improved by the isotype switching [67,72].

On the other hand, the domain-domain associations need to be taken into account when studying the aggregation of IgG, which contains substantial molecular-scale interactions in the V_H_/V_L_, C_H_1/C_L_, C_H_2/C_H_3 and C_H_3/C_H_3 interfaces. The domain-domain interactions confer IgG interface free energy for native folding, which would disappear when one of the interacting domain unfolds [57]. Thus, the unfolding and aggregation of full length IgG is often triggered by the least stable domain. It is believed that C_H_2 unfolds first and triggers the aggregation process, while C_H_3 is the most stable domain [73]. Fab usually bears the middle thermostability, but is significantly modulated by the CDR sequences [74]. IgG aggregation is most frequently studied at an acidic pH since IgG needs to be exposed to acid for resin purification or virus clearance [35]. The low pH renders the charged residues in the domain interfaces experiencing protonation, which could disturb the hydrogen bonds and salt bridges [59]. Consequently, the polar residues would carry excess charges and destabilize IgG by intramolecular charge–charge repulsions [7]. The low pH was often combined with temperature ramping for evaluating enthalpy changes and the unfolding intermediates during IgG unfolding determined by DSC measurement [57]. The DSC thermograms of IgG usually contain more than one endotherm peak corresponding to the unfolding of its separated domains [73,75], which indicates that the thermostability of the individual domain collectively contributes to the overall aggregation propensity of full-length IgG. Therefore, to explore the strategies for mitigating IgG aggregation, one could scrutinize the factors influencing aggregation on the basis of its separated domains. Studying the aggregation of antibody domains is more straightforward and informative. The aggregation-resistant lessons learned from single or multi-antibody domains could guide the aggregation-resistant engineering of full-length IgG. Therefore, studying the aggregation of antibody domains or fragments has significant relevance for combating the aggregation of full-length IgG. In the next section, we will discuss the relationships between aggregation propensity and the structures of antibody domains, as well as their aggregation-resistant strategies.

### 3.3. V_H_ and V_L_

**Figure 4:**
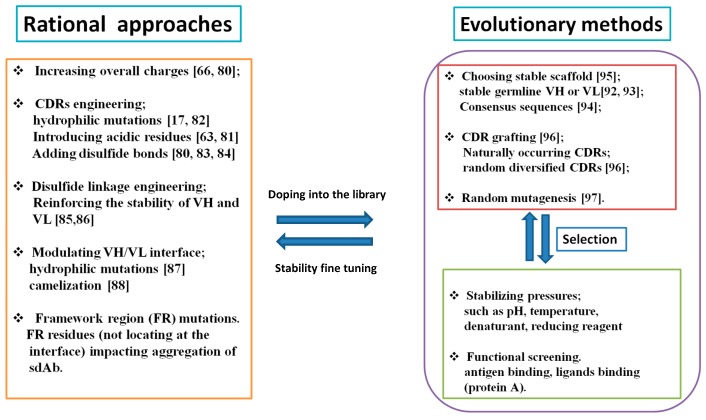
Rational approaches and evolutionary methods for decreasing the aggregation of human single-domain antibodies. These two methods could be combined to develop aggregation-resistant V_H_ or V_L_. The rationally-designed mutations can be doped into the library to increase the possibility of identifying binders with superior biophysical properties. The binders obtained from such a library could be subjected to stability fine-tuning and further guided by rational approaches.

V_H_ and V_L_ as single domains are the smallest units for antigen binding. These single domains have attractive advantages as diagnostic and therapeutic reagents due to their small size, which confer them capacity to access cryptic epitopes and enhance penetration into solid tissues [76]. V_H_ and V_L_ are intrinsically prone to aggregation due to the exposure of the hydrophobic V_H_–V_L_ interface. V_H_ has been shown to be more prone to aggregation than V_L_, and its aggregation is more dependent on its CDRs than V_L_ [77]. The stabilizing free energy for V_H_ and V_L_ results from the hydrophobic core inside the immunoglobulin β barrel and the disulfide bond connecting the two β sheets layers. While an intra-domain disulfide in the antibody domain contributes a range of 4–6 kcal/mol of free energy to the folding [78], 1 Å^2^ of the hydrophobic contact corresponds to 25 cal/mol [79]. The CDRs responsible for antigen binding also impact the colloidal stability and aggregation of V_H_ and V_L_. Generally, the aggregation resistance strategies could be divided into rational and evolutionary approaches. While rational approaches exploit structure-based knowledge or sequence information to guide the aggregation-resistant mutations, the evolutionary methods involve the selection of a single domain antibody library by stability-improving pressures, such as temperature and pH. These two approaches could be combined to design aggregation-resistant single domain antibodies (sdAb) (Figure 4).

The elucidation of structural and sequence determinants underlying aggregation propensity diversifies the rational approaches of combatting the aggregation of sdAb, which include the engineering of the overall charge, CDRs, disulfide bonds, V_H_–V_L_ interface and the residues in framework (FR) regions influencing aggregation. Antibodies with net charges are less prone to aggregation due to the intermolecular repulsions. Tanha et al. have reported that the aggregation-resistant human V_H_s obtained by panning against target enzymes using a transient heat denaturation approach bear acidic pIs, similar to the naturally-occurring camelid V_H_H and shark V_NAR_, which is reminiscent of protein acidification constituting a universal mechanism to confer functional sdAbs [80]. The same principle may be also applied to the full-length IgG. Schaefer et al. have shown that IgG expressed in yeast bearing the “EAEA” sequence at the N termini of the light chain and heavy chain are more aggregation resistant than the counterpart expressed in mammalian cells [66]. Consistent with this, introducing the negatively-charged residues into CDRs is a feasible route for mitigating the aggregation of V_H_ and V_L_. Perchiacca et al. have improved the solubility of V_H_ containing aggregation-prone Aβ peptides by inserting the “DED” triad into the edge of CDR3 [81]. Dudgeon et al. have found that incorporations of negatively-charged residues, especially Asp, into the CDR1 of V_H_ (Positions 28, 30–33, 35) and CDR2 of V_L_ (49, 50–53, 56), are universal strategies for conferring aggregation resistance to sdAbs [63]. In addition to the introduction of negatively-charged residues, CDRs are subjected to extensive aggregation-resistant engineering since CDRs are hydrophobic “hot spots” mediating the aggregation of sdAbs. Rational designs usually include the mutagenesis of hydrophobic residues into hydrophilic ones [17,82] and the introduction of cysteines into CDR3 to constrain the conformation of the long protruding CDR3 or to mediate cross-linking with CDR1 to stabilize sdAbs [80,83,84]. On the other hand, the exogenous disulfide bond could be introduced into the FR regions to strengthen the thermostability of V_H_ and V_L_. Kim et al. have reported that the introduction of the non-canonical disulfide bond between Cys54 and Cys78 residues increased the thermostability of V_H_ by 14–18 °C [85]. In addition, the same authors have also achieved improving the thermostability of V_L_ by 5.5–17.5 °C through the creation of disulfide bonds linking residues 48 and 64 [86]. Another aggregation-resistant method involves the engineering of the exposed V_H_–V_L_ interface that would otherwise be buried in intact antibodies or other bigger fragments. Indeed, hydrophilic mutations near the former V_H_/V_L_ interface have been demonstrated to improve the solubility of dAbs [87]. The naturally-occurring V_H_H from camels has inspired the screening of aggregation-resistant mutants of V_H_, although the camelized mutations are often not applied to therapeutic mAbs considering the potential immunogenicity [88]. Furthermore, accumulating research has established the impact of the residues in FR regions on the aggregation of sdAbs. For example, the residues of Glu or Gln6 [89], Arg66 and Gln105 [90] in V_H_ and residues of R24, Y49 [63] and Pro8 [91] in V_L_ are the key players in mediating the aggregation of sdAbs.

Alternatively, the library-based evolutionary approaches could be used for alleviating the aggregation of sdAbs. Antibody domains of different sub-families bear distinct thermostability and aggregation propensity. The V_H_1, 3 and 5 gene families generally displayed better physical characteristics than the 2, 4 and 6 gene families [92]. For V_L_, V_κ_ is more desirable than the V_λ_ chain, with V_κ_3 being the most thermodynamically-stable followed by the V_κ_1 and V_κ_4 [93]. Besides, based on the sequence and structural comparisons, some groups have found the so-called “consensus sequences” benefiting the folding of V_H_, which usually bear high frequencies in the repertoire [94]. By combing the favorable germline domains and those consensus sequences, one could establish stable scaffolds to construct libraries with randomized CDRs, which could be further subjected to some pressures to select the biophysically-desired binders [95]. Our group succeeded in isolating a stable V_H_ antibody, m36, for targeting HIV-1 CoRbs by panning a V_H_ phage library, which is composed of a stable scaffold based on the V_H_3 with the incorporation of partially-randomized CDR1 (Positions 30 and 31) and naturally-occurring CDR2 and CDR3 [96]. On the other hand, one could perform random mutations for the selection of stability-improved sdAbs, in the case where the parent sdAb for a given antigen has already been developed [97].

### 3.4. scFv

scFv consists of the variable domains of V_H_ and V_L_ connected by a flexible linker, which combines with the strong inter-domain associations to ensure that scFv retains the antigen binding of the full-length IgG if no reorientation of the V_H_ and V_L_ occurs. Due to its small size (~30 kD), scFv bears many therapeutic merits as a diagnostic and therapeutic agent, such as easy expression, relative stability compared to V_H_ and deceased toxicity when used in radioisotopes and bio-imaging [98]. Like the separated V_H_ and V_L_, the thermostability and aggregation liability of scFv relates tightly to the CDRs’ sequences and the intrinsic stabilities of the sub-domains [99]. Thus, the general aggregation-resistant strategies for V_H_ and V_L_ are also applied to the scFv. General strategies include introducing the stabilization mutations, such as residues 6 and 66 in V_H_, Pro8 in V_κ_ [89,91], choosing stable frameworks of V_H_ and V_L_, such as the combinations of H3_κ_3, H1b_κ_3, H5_κ_3 and H3_κ_1 for constructing scFv [92] and introducing CDR mutations (see the above *V_H_ and V_L_* Section). However, as a fragment with strong V_H_/V_L_ associations, scFv has its unique features. The V_H_/V_L_ interface of scFv is usually conformationally dynamic and experiences “transient opening”, which could expose the hydrophobic patches to promote aggregation [100]. Therefore, stabilizing the V_H_–V_L_ interface has enjoyed many successes for improving the solubility of scFv. Corresponding strategies include introducing a disulfide bond into the contact interface [101], forming new salt bridges and hydrogen bonds between V_H_–V_L_ [102] and adding “knob-into-hole” mutations [103]. Another influential factor impacting scFv aggregation is the monomer-dimer-multimer equilibrium occurring via the domain-swapping manner, where the V_H_ domain of one scFv pairs with the V_L_ domain of another scFv and vice versa [104]. This domain swapping highly depends on the linker length in which the short linker of 5–10 residues forms a stable dimer (diabody), and the long linker of 15–20 amino acids favors a monomer [99]. Thus, optimizing the linkers between V_H_ and V_L_ by changing both the linker sequences and length could alleviate the aggregation of scFv [105]. Including linkers of 25 amino acids is reported to be a common way of increasing the stability of scFv by populating monomer species [99]. Besides, the domain swapping is also impacted by the expression condition, solution ionic strength and pH [106].

### 3.5. Fab

Fab contains four domains, namely, V_H_, C_H_1, V_L_ and C_L_, in which V_H_/V_L_ and C_H_1/C_L_ strongly associate via hydrophobic contact and hydrogen bonding, while few interactions exist at the V_H_/C_H_1 and V_L_/C_L_ interface. Fab has unique advantages as the diagnostic tool and pharmaceutic reagent due to its small size, easy production and relatively longer in vivo half-life than the single domain antibody [107]. Fab-based therapeutics is currently on the market, e.g., ReoPro (Centocor), Lucentis (Genetech) and Cimzia (UCB) [108]. Generally, Fab is more stable than V_H_ and scFv since the hydrophobic interfaces exposed in V_H_ and scFv are buried by the constant domain in Fab. Like sdAbs and scFv, the unfolding and aggregation propensity of Fab are also impacted by CDRs. It is reported that IgG pools from human blood exist as dimers due to the association of the distal ends of their Fab arms, similar to the idiotype-anti-idiotype complexes [109]. The properties of CDR residues could modulate the colloidal stability, thus mediating the self-association of Fab in the native condition [110]. Besides CDRs, the sequences and structural features of FR regions also impact colloidal interactions by altering the charge distributions across the Fab [20]. Meanwhile, colloidal interaction could be deeply modulated by the solution pH and ionic strength [108]. Another factor complicating Fab aggregation is the inter-domain disulfide bond. The disulfide bond at the C terminus of C_H_1 and C_L_ of IgG1 can lock domain conformations and strengthen the inter-domain interactions. By mimicking this disulfide bond, Peters et al. have improved the thermostability of Fab and the disulfide bond heterogeneity of full-length IgG4. This disulfide bond was formed through mutating C127 in the N terminus of C_H_1 to a serine and simultaneously introducing a cysteine at the C terminus of C_H_1 (Positions 227–230), which facilitates disulfide bonding to the C_L_ [111].

**Figure 5:**
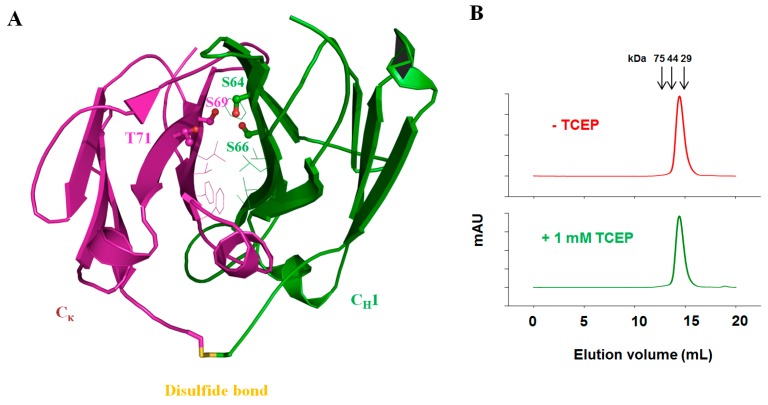
Rational design and identification of stabilized C_H_1–C_κ_. (**A**) Structural analysis of the C_H_1–C_κ_ interface. The side chains of hydrophobic residues at the interface are shown in slim stick representation. The four amino acid residues lining a void structure are indicated with their side chains shown in a bold ball-and-stick representation; (**B**) Size-exclusion chromatography of mD1.22-C_H_1/m36.4-C_L_ variants. Proteins were treated with and without 1 mM TCEP (tris(2-carboxyethyl)phosphine) before analysis. The arrows at the top indicate the elution volumes of the molecular mass standards in PBS (pH 7.4): carbonic anhydrase (29 kDa), ovalbumin (44 kDa) and conalbumin (75 kDa).

On the other hand, unlike sdAbs, Fab structurally features two inter-domain interactions, which are targets for aggregation-resistant strategies. The domain-domain cooperativity of V_H_/V_L_ and C_H_1/C_L_ in Fab is highly predisposed in the unfolding process, as shown by the single and sharp endotherm peaks in the DSC thermograms [57]. The C_H_1/C_L_ interface is believed to be more stable than the V_H_/V_L_ interface, but also depends on the characteristics of CDRs. While the V_H_/V_L_ interface is pre-defined for a given Fab, which has limited spaces for engineering considering the requirement of attaining the antigen binding, the C_H_1/C_L_ interface constitutes a convenient platform for improving inter-domain interactions. Teerinen et al. have reported that the solvated hydrophobicity of C_H_1/C_L_ could be increased by mutating Thr178 to Val or Leu, which leads to increased unfolding free energy of Fab [112]. Recently, our group has successfully used an evolutionary method to introduce a set of mutations in the C_H_1/C_L_ interface including S64E, S66V in C_H_1 and S69L, T71S in C_κ_. These mutations conferred C_H_1/C_L_ with improved inter-domain associations due to enhanced hydrophobic contact and hydrogen bonds [113]. The improved variant could retain its homogenous monomeric state even in the presence of 1 mM TCEP, indicating that the lack of disulfide bond has no impact on the non-covalent inter-domain interactions (Figure 5).

### 3.6. Fc

The hydrophobic regions contain APRs promoting antibody aggregation. For IgG, more APRs are found in Fc than in Fab, as evidenced by the molecular simulations performed by Bernhardt et al. [53]. By using the so-called SAP method, they have found as many as 14 aggregation-prone motifs in the IgG1 constant domains. These motifs contain one to seven residues and are largely conserved among all IgG subclasses (IgG2, IgG3 and IgG4). According to their research, most of those motifs are located at the lower hinge region and C_H_2–C_H_3 interface of Fc. Indeed, the hydrophobic lower hinge (sequence: 231-APELLGGPSVFLFPP-245) is not only the “hot” site for engineering IgG effector function, such as antibody dependent cell mediated cytotoxicity (ADCC) and complement dependent cytotoxicity (CDC) [68], but also a focus for improving antibody solubility and thermostability. In Bernhardt et al.’s later study, the authors have improved the solubility of full-length IgG by mutating the hydrophobic residues of L234 and L235 into lysine. This improvement probably results from the enhanced thermostability of C_H_2 domain as revealed by the DSC analysis [54]. Gong et al. have achieved improving the thermostability of the C_H_2 domain and decrease aggregation propensity by removing the unstructured loop composed of sequence of N-APELLGG-C [114]. Furthermore, the same authors have found that the aggregation of C_H_2 could be significantly decreased by mutating APRs residues, such as F241, F243 identified by the TANGO program into hydrophilic residues (personal communications). Another hot spot for combating aggregation lies at the C_H_2–C_H_3 interface, which bears high adaptability to binding different ligands, such as protein A/G, FcRn and rheumatoid factor [115]. DeLano et al. have shown that the C_H_2–C_H_3 junction contained a hydrophobic consensus motif composed of Asn434, Ile253, Met423, Tyr436, Met252 and Ser254 for accommodating different natural scaffolds, such as two α-helices of the B-domain of protein A and two β-strands (engineered peptide with sequence of DCAWHLGELVWCT) [115]. By mutating I253 to lysine, the solubility and stability of full-length IgG1 could be improved according to Bernhardt et al.’s study [54]. Another elegant piece of evidence clarifying the implications of this hydrophobic patch in IgG aggregation came from Kolenko et al.’s research, showing that, in the crystal structure of mouse IgG2b-Fc, the C_H_2–C_H_3 interface interact with the C’E loop (the residues Arg293–Thr299) containing glycosylated Asn297 and another nearby loop of the same neighboring molecule [116]. This complex of two antiparallel-oriented dimers of the Fc provided a structural model of Fc:Fc-mediated immunocomplex (IC) formation and increased aggregation. The involvement of the C’E loop in Fc–Fc association may also have relevant for the roles of glycans in the stabilization and aggregation of IgG. Chen et al. have observed enhanced thermostability and decreased aggregation of full-length IgG1 after introducing an engineered aromatic sequon (EAS) (Q295F/Y296A) into the glycosylated C’E loop [117]. Although the aggregation resistance was ascribed to the improved stability of C_H_2 due to the interactions between F295 and GlcNAc1 and core fucose, it may be interesting to explore how the altered C’E loop impacts the Fc–Fc interactions and subsequent colloidal aggregation of IgG1.

Fc aggregation depends on the thermostability of C_H_2 and C_H_3. Actually, C_H_2 is considered to be the least stable domain and usually triggers the unfolding of full-length IgG [118]. Therefore, improving the thermostability of C_H_2 and C_H_3 could benefit the stability and solubility of the full-sized IgG. One effective strategy for improving the stability of C_H_2 and C_H_3 is modulating intra-domain disulfide bonds. Gong et al. have increased the transition melting temperature (T_m_) of C_H_2 from 54.1 °C to 73.8 °C by introducing a disulfide bond in Positions 242 and 334 (m01) [119]. Ying et al. have achieved improving the T_m_ of a monomeric C_H_3 from 41 °C to 76 °C by introducing a disulfide bond connecting Positions 343 and 431 [120]. Wozniak-Knopp et al. have reported that the human IgG1 Fc could be stabilized by introducing intra-domain disulfide bonds in the C_H_3 domain. In their research, two engineered disulfides with one linking the N-terminus of the C_H_3 domain with the F-strand and the other connecting the BC loop and the D-strand collectively led to an increase of the T_m_ of ~15 °C for IgG Fc [121]. Collectively, Fc with widely-distributed APRs has gained much attention for aggregation resistance, which benefits the design of full-length IgG with improved biophysical properties.

## 4. Aggregation of Antibody Drug Conjugates

Antibody drug conjugates (ADCs) combine the specificity of monoclonal antibodies (mAbs) with the potent cytotoxic activity of small molecule drugs for the treatment of cancer and other diseases [122]. The development of ADCs has been significantly hampered due to the strong tendency of ADCs to aggregate or “clump up” [123].

ADCs have three major components, antibody, linker and the cytotoxic drug as the payload. Clearly, the aggregation propensity of ADC is directly related to the parent mAbs, as exemplified by Beckley et al. showing that eight kinds of ADCs with the same linker-payloads of vc-MMAE (monomethyl auristatin E), but with distinct variable domains had different propensities of forming high molecular weight species (HMWS) [124]. Meanwhile, the aggregation of ADC is complicated by the conjugation chemistry and the nature of linker-payloads. The conjugation reaction usually involves the activation of side chains of cysteine, lysine or the keto group of carbohydrate from mAb and subsequent ligation with functionalities from the linkers. During the production process, the activated intermediates containing free sulfhydryl group of Cys and the maleimido group from the linker could mediate the inter-molecular crosslinking and lead to aggregation. Wakankar et al. have demonstrated that a lysine-activated intermediate, Tmab T-MCC (Trastuzumab-maleimidylmethyl cyclohexane-1-carboxylate), was more prone to aggregation than the fully-conjugated ADC T-DM1 (Trastuzumab emtansine) [125]. Additionally, the conjugation type could impact the conformation of mAbs and alter aggregation potentials. It was reported that thiol ligation could induce more aggregation than lysine coupling [126], probably due to the reduction of the inter-chain disulfide bonds in thiol coupling.

**Figure 6:**
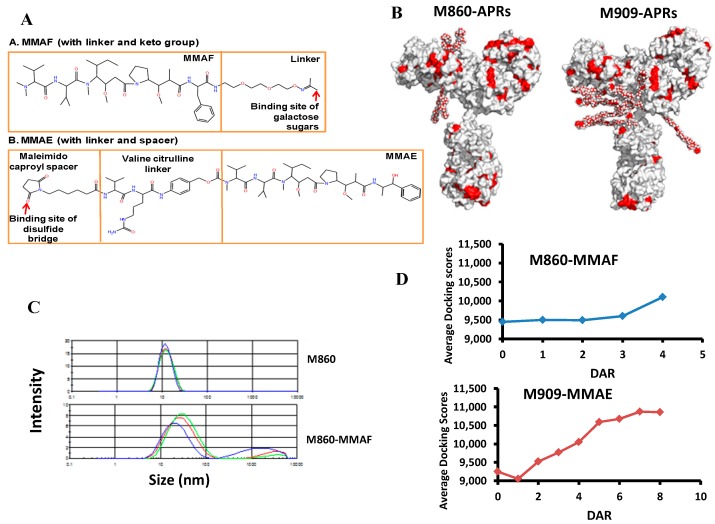
The aggregation of ADC. (**A**) The chemical structures of monomethyl auristatin F (MMAF) and E (MMAE) and their respective linkers and spacers; (**B**) The molecular surface diagrams of antibody drug conjugates (ADCs) with the predicted aggregation-prone regions (APRs) colored in red. Homology models of the ADCs M860-MMAE and M909-MMAF have the maximum number of four drugs for M860-MMAF and eight drugs for M909-MMAE. The APRs of the antibody are the consensus residues predicted by four combinations of calculation programs, Aggrescan, TANGO, WALTZ and Amylpred2. The APRs of the drug molecules are the nonpolar carbon atoms; (**C**) The aggregation tendencies of M860 mAb and M860-ADC measured using the dynamic light scattering test to determine the size distribution profile after seven days of incubation at 4 °C and 37 °C (1 mg/mL of PBS buffer). (**D**) The docking scores of M860-MMAF and M909-MMAE with drug-antibody ratios (DARs). The average docking scores of M860-MMAF and M909-MMAE were simulated and calculated using PatchDock.

For the impact of the linker-payload, their hydrophobically-aliphatic moieties expand the APRs on ADC, facilitating the aggregation by forming hydrophobic patches. Guo et al. have pointed out the contributions of linker payload to the overall hydrophobicity of the ADC by molecular modeling [127]. The increased hydrophobicity of ADC mediated by the linker payload is also supported by the hydrophobicity calculations and APR predictions of two ADCs developed in our group, m860-monomethyl auristatin F (MMAF) and m900-MMAE. M860-MMAF is a sugar keto conjugation ADC targeting the HER2 receptor [128], and m909-ADC is a thiol conjugation targeting the folate receptor β [129] (Figure 6A). Their APRs were predicted by Aggrescan, TANGO, WALTZ and Amylpred2, and the hydrophobicity was empirically calculated by measuring the non-polar surface area of both antibodies and drug molecules [130]. Results showed that linker payload contributed to APRs, as well as the overall hydrophobicity (Figure 6B). Due to the increased hydrophobicity, ADCs are more prone to aggregation compared to the parent mAbs. Guo et al. have reported that an ADC with a maleimidocaproyl linker and an auristatin payload are more prone to aggregate during thermal stress than the parent mAb, although they have similar secondary and tertiary structures [131]. Similar results were obtained by our group for m860-MMAF (Figure 6C). To reduce the hydrophobicity of ADC, some groups have studied replacing the hydrophobic linkers with hydrophilic linkers or PEGylation linkers. Zhao et al. have incorporated sulfonate- or PEG-containing hydrophilic linkers into antibody maytansinoid conjugates to achieve high DAR without aggregation and low non-specificity [132]. Lyon et al. have exploited a hydrophilic glucuronide linker in PEGylated ADCs to decrease the hydrophobicity of ADC and to extend its in vivo half-life [133].

The one-pot chemical conjugation of mAb and drug reactants usually produces heterogeneous ADC products. In the case of thiol-maleimide chemistry, the extent to which the inter-chain disulfide bonds were reduced determined the quantity of drugs attaching to mAb, which led to the ADC species with different DARs ranging from 0 to 8. DAR constitutes a major concern for designing ADC therapeutics, since DAR tightly impacts the aggregation propensity, in vivo potency and serum stability of ADC. ADC with high DAR usually has higher aggregation propensity due to the increased hydrophobicity conferred by the hydrophobic drug molecules. Guo et al. have found that their ADC with DAR6 species may exist in a multimeric state, while DAR2 and DAR4 species likely exist in monomeric forms under ambient conditions [127]. Beckley et al. have reported that their ADC aggregate mainly contained the high DAR species of 6–8 [124]. Meanwhile, ADC with high DAR was reported to be subjected to more structural perturbations, causing the destabilization of mAb, as exampled by Adem et al. showing that high DAR species readily experienced aggregation and fragmentation under stress conditions, such as high ionic strength buffer, due to the fewer inter-chain disulfide bonds [134]. The destabilized structure of high DAR species could probably reconcile its fast plasma clearance [133] when encountered with protease degradation. Besides, Pan et al. have reported that ADC bears distinct conformation at the C_H_2–C_H_3 interface compared to the parent antibody [135], which may disturb its interaction with FcRn and impact the serum half-life of ADC. In addition, the authors also found that the low hinge region of the C_H_2 domain became more solvent exposed in ADC than in the naked mAb by using hydrogen/deuterium exchange mass spectrometry (HDX-MS). The conformation alteration of C_H_2 domain was also confirmed by Beckley et al., showing that the C_H_2 domains in ADC with high DAR bear low stability and rapidly form aggregates at 40 °C [124]. The fact that the higher DAR could pose more structural alterations is further supported by our molecular docking (PatchDock simulations [136]) studies of m860-MMAF and m900-MMAE with different DARs. Our results showed that the propensity of ADCs associating with each other (docking score) positively correlated with the DAR (Figure 6D), indicating that the higher DAR species presents a conformation that is more prone to associate with each other.

Collectively, the aggregation of ADC is a function of the naked antibody, linker payload, conjugation chemistry and DAR, as well as the formulation solution, all of which need to be considered when trying to increase the aggregation resistance of ADCs.

## 5. Conclusions and Outlooks

The aggregation of antibody domains has been extensively studied in order to expedite the screening of aggregation-resistant IgG in the early development process. However, caution is needed since aggregation resistance strategies for antibody domains are not always successfully transferred onto full-sized IgG. Usually, the strategies for improving the stability of the constant domain could be applied to full-length IgG. For example, our group has found that the stabilization mutations at the C_H_1/C_κ_ heterodimer interface (S64E/S66V of C_H_1 and S69L/T71S of C_κ_) identified from the Fab-like format could be applied onto the full-length IgG-like format (4Dm2m) [113]. Kelly et al. have found the mutations of Q295F/Y296A stabilizing the C_H_2 domain through interaction with glycan could also confer full-sized IgG decreased low pH-induced aggregation [117]. By contrast, transferring of aggregation properties from variable domains to full-sized IgGs is often complicated by the antigen binding and domain-domain associations. Pepinsky et al. have reported that the anti-LINGO-1 Mab Li33 selected from the Fab phage library had poor solubility when converted into IgG1 format [67]. Another example came from Daniel Christ’s group, showing that the aggregation resistance benefit derived from mutating residues in CDR1 of V_H_ and CDR2 of V_L_ into negatively-charged residues, such as Asp and Glu, could successfully be transferred into scFv format [63]. The scFv trastuzumab with double mutations of 30D/52D not only resisted aggregation, but also retained high binding affinity to HER2. Furthermore, these mutations have been incorporated into the full-length trastuzumab IgG1 without disturbing the antigen binding and the biological functions. However, it remains to be seen whether these mutations alleviating aggregation of the single domain antibody could also improve the solubility of the full-length IgG1.

One should also keep in mind that the same forces promoting protein aggregation also operate in protein folding and interactions. As such, one needs to balance between decreasing aggregation and maintaining the correct folding and specific binding when rationally-designing aggregation-resistant antibodies. In our development of aggregation-resistant m36.4 variants, we have found that although some mutants indeed gained decreased aggregation according to the dynamic light scattering (DLS) profiles, their unfolding T_m_s was significantly compromised as measured by the temperature ramping CD spectra. Meanwhile, mitigating aggregation through engineering the IgG sequence and structure by mutagenesis bears risks to generate new B- and T-cell epitopes and to cause immunogenicity. While the mutations occurring at CDRs could induce an anti-idiotype response and lead to the neutralization of the therapeutics, mutations at the constant domain could evoke the “binding antibodies” response in the host and compromise the efficacy of therapeutics due to the changed pharmacokinetics [137]. Thus, the risks of eliciting immunogenicity should be carefully considered when designing aggregation-resistant mutations, although many CDR mutations are reported to cause little or no immunogenicity in clinical trials [138]. An effective approach to limit immunogenicity is germlining to the human counterpart. Thus, the mutations of IgG sequences should be as few as possible. Fortunately, the potential of introducing T-cell epitopes by mutations could now be predicted in silico due to the mapping of their interactions with the human leukocyte antigen (HLA) class II molecules [139]. Besides, one should pursue exploiting the benefit of the hydrophilicity of the carbohydrate and PEG (polyethylene glycol) to combat the aggregation propensity of IgG, since glycosylation and PEGylation of IgG have a much decreased possibility to induce immunogenicity [140,141].

The antibody multi-domain nature defines its aggregation process as pathway dependent, which is a function of a combination of IgG sequences and structures, as well as the solution conditions. While most designs focus on improving thermostability and alleviating the aggregation of antibody fragments in a lumped assay, such as turbidity and dye binding, few studies are dedicated to clarifying to what extent the engineering strategies impact the formation of the aggregate intermediates or the soluble irreversible aggregate species. Similarly, few existing computational programs account for the aggregation mechanism of the antibody although they could predict APRs in antibody sequences. Furthermore, calculation tools have failed to incorporate the external solution condition into the aggregation prediction. Although several programs have indeed considered the physiological conditions in the prediction, therapeutic antibodies are not usually produced, stored and administered under physiological conditions. Thus, these calculation methods poorly learn the antibody aggregation-resistant engineering in some cases. The studies of soluble aggregate intermediates, aggregation kinetics and mechanisms, as well as their dependence on the solution conditions would guide the antibody formulation optimization, which should be extensively pursued in future research work.

In summary, the aggregation propensities and aggregation-resistance strategies for antibody domains have been extensively investigated. Future studies are worth understanding the aggregation mechanisms for full-sized antibodies and their dependence on the environment, which could expedite antibody therapeutics development.
